# MDCL-DETR: Multi-Domain Enhancement and Cross-Layer Feature Fusion for Small Object Detection

**DOI:** 10.3390/s26113305

**Published:** 2026-05-22

**Authors:** Tianran Hao, Xiao Zhang, Bing Zhou

**Affiliations:** School of Computer Science and Artificial Intelligence, Zhengzhou University, Zhengzhou 450001, China; iehaotianran@gmail.com (T.H.); iebzhou@zzu.edu.cn (B.Z.)

**Keywords:** multi-domain enhancement, feature fusion, small object detection, uncrewed aerial vehicle (UAV)

## Abstract

Small object detection in uncrewed aerial vehicle (UAV) imagery is hindered by limited pixels, insufficient detailed information, and strong background interference, leading to weak feature representation and poor contextual modeling. To address these issues, we propose a multi-domain enhancement and cross-layer feature fusion detection Transformer (MDCL-DETR) with progressive feature processing. First, a multi-domain enhancement module (MDEM) based on CSP (cross stage partial) structure is proposed, which fuses spatial and frequency-domain features in a lightweight manner to enhance object detail and global structures while effectively distinguishing object features from background interference. Second, a cross-layer feature extraction module (CLEM) is introduced to aggregate multi-scale features across layers, alleviate information loss caused by downsampling, and preserve spatial details of small objects while integrating high-level contextual semantics. Meanwhile, a gated Mamba fusion module (GMFM) is proposed, which adopts the Mamba architecture for long-range dependency modeling of multi-scale features and integrates a gating mechanism to realize the dynamic weighted fusion of local details and global context, further improving feature discriminability and global modeling capability. Finally, a fine-grained enhancement module (FGEM) is designed, which leverages feature reorganization and adaptive feature extraction to reinforce and compensate fine-grained features. Extensive experimental results validate the effectiveness and generalization of the proposed method, achieving mAP_50_ scores of 54.1% and 56.2% on the VisDrone2019 and AI-TOD datasets.

## 1. Introduction

UAVs have been widely applied in military reconnaissance [1,2,3], urban construction [4], agricultural management [5], and other fields [6]. UAV-based object detection has become an important research direction in computer vision. However, small objects in aerial images occupy only a small number of pixels, with scarce feature information and high vulnerability to interference from complex backgrounds, which significantly increases the difficulty of detection.

To address the challenges of small object detection, researchers have proposed various technical approaches. One-stage detectors, represented by YOLO [7,8,9] and SSD [10], are favored for their high efficiency, and they have alleviated the detection problem of objects at different scales to a certain extent by introducing multi-scale feature fusion structures such as FPN [11] and PANet [12], but they perform poorly in small object detection. In recent years, the Transformer architecture has achieved remarkable progress. Carion et al. [13] first proposed DETR, a Transformer-based object detection algorithm, which simplifies the detection pipeline by removing the NMS post-processing operation and adopting learnable object queries to directly predict categories and bounding box coordinates. However, DETR performs poorly in small object detection. To solve this problem, Zhu et al. [14] proposed Deformable-DETR, which introduces multi-scale features and encoding at different levels to improve small object detection accuracy. DQ-DETR [15] designed a technology-guided feature enhancement module to strengthen the spatial information of small objects. The Baidu team [16] proposed RT-DETR, which introduces a hybrid encoder to effectively fuse multi-level features and improve the capture capability of detailed information in images. Li et al. [17] designed frequency-domain self-attention and mutual auxiliary channel attention, achieving efficient global feature enhancement in the frequency domain and completing cross-level feature mutual fusion. Lin et al. [18] adopted a wavelet-based frequency-domain auxiliary enhancement strategy to preserve high-frequency details and strengthen object edges. Small objects occupy only a limited number of pixels, and their discriminative features such as textures and contours are highly susceptible to loss during downsampling. By separating high-frequency details from low-frequency background components, frequency-domain representation can precisely preserve and enhance the edge and texture information of small objects. Effectively capturing discriminative features by collaboratively exploiting spatial and frequency-domain information remains a challenging research issue. In addition, how to fuse complementary information at different layers and capture discriminative features is also a valuable research topic.

In this paper, we propose an improved real-time object detection algorithm based on RT-DETRv2 [19], named MDCL-DETR: a multi-domain feature enhancement and cross-layer feature fusion-based object detection model. This framework incorporates four core modules for progressive feature extraction. Specifically, a multi-domain enhancement module (MDEM) is first employed to distinguish objects from background noise. Subsequently, a cross-layer feature extraction module (CLEM) preserves spatial details while integrating high-level semantic information. A gated Mamba feature fusion module (GMFM) then achieves dynamic weighted fusion of multi-scale features, and finally, a fine-grained enhancement module (FGEM) reinforces and compensates for the fine-grained features of small objects. Operating in a progressive, layer-wise manner, these modules iteratively optimize features from basic representations to high-level semantics, thereby enhancing the model’s perception capability for small objects. Extensive experiments are conducted on the publicly available small object detection datasets VisDrone2019 and AI-TOD, which validate the effectiveness and superiority of the proposed framework. Overall, the main contributions are summarized as follows:

1. We propose a CSP-based MDEM for lightweight spatial frequency domain feature fusion, which alleviates small-object feature attenuation, enhances detail and global representation, suppresses background interference, and provides high-quality features for multi-scale fusion.
2. We introduce a CLEM that employs dilated convolution to fuse multi-level features, addressing the imbalance between semantic and detailed information and effectively capturing multi-scale contextual information.
3. We design a GMFM that integrates gating units with Mamba structure modeling to tackle feature redundancy, noise, and insufficient contextual dependency modeling after multi-scale fusion, dynamically filtering valid features, capturing long-range feature dependencies, strengthening global information correlation, and improving feature discriminability and global modeling capability.
4. We develop an FGEM that reorganizes and dynamically fuses shallow spatial features to compensate for the fine-grained features of small objects, reinforcing object details and edge information.

## 2. Related Work

### 2.1. Small Object Detection

Small object detection faces extremely challenging problems, as small objects are characterized by small sizes and sparse, weak feature information, leading to a significant increase in detection difficulty. To address this challenge, small object detection methods can be roughly divided into three categories. The first direction is data augmentation. For instance, Kisantal et al. [20] enhanced the diversity of small objects by rotating, copying, and randomly distributing them to arbitrary positions in the image; RRNet [21] introduced an adaptive data augmentation strategy, which employs a pre-trained semantic segmentation network to generate a prior segmentation map for guiding the pasting positions of small objects. The second direction is feature enhancement. For example, Cui et al. [22] designed a resolution restoration decoder to enhance the degraded information of small objects; Zhang et al. [23] enhanced the edge features of small objects using channel and spatial attention, and introduced a multiple aggregation feature pyramid network to enrich the semantic information of shallow features, thus improving the performance of small object detection. Gao et al. [24] introduced subpixel super-resolution features to enhance the representation capability of small objects, and designed a cross-scale distillation method to focus on the features of small-object regions. Zhu et al. [25] proposed hybrid feature enhancement to improve the representation capability of semantic features for small objects. Zhang et al. [26] designed SR-CLIP, which fully captures contextual information by fusing text information with image features. Zhang et al. [27] proposed the Dynamic Feature Extraction Module and the Adaptive Downsampling Module to enhance the contextual information and local details of tiny objects. The third direction is multi-scale feature fusion. Lin et al. [11] proposed a feature pyramid network (FPN), which fuses multi-scale features using a top-down architecture with lateral connections. Yang et al. [28] proposed a method that integrates multiple attention mechanisms into residual networks to achieve multi-scale feature fusion, thereby improving the detection accuracy of small objects. Liu et al. [29] proposed DNTR, which utilizes contrastive learning and self-attention mechanisms to suppress noise at different levels and enhance the feature representation of tiny objects. Liu et al. [30] designed the RepVit block and RepVit downsample to extract multi-scale features, maintaining competitive performance even under a lightweight network structure.

Based on the above research on small object detection, effective feature enhancement and fusion are beneficial to improving the performance of small object detection. However, the existing methods mainly focus on spatial-domain feature extraction and neglect the utilization of frequency-domain features. Our work focuses on enhancing the representation of small objects through spatial frequency domain features and captures multi-scale information by effectively fusing cross-layer features, so as to improve the detection performance of the model.

### 2.2. Transformer-Based Object Detection

The Detection Transformer (DETR) was first proposed by Carion et al. [13], and innovatively combines a Transformer with a CNN, eliminates non-maximum suppression (NMS) and region proposal generation processes, and uses object queries to directly predict object categories and bounding box coordinates, achieving end-to-end detection. However, DETR suffers from an excessively long training cycle and poor performance in small object detection tasks. Zhu et al. [14] proposed Deformable DETR, which effectively captures objects at different scales by introducing a deformable attention mechanism. Li et al. [31] proposed DN-DETR, which improves detection performance by introducing noisy labels during the training process. Meng et al. [32] proposed Conditional-DETR, Liu et al. [33] proposed DAB-DETR, and Wang et al. [34] proposed Anchor-DETR; these methods embed bounding box position and scale as learnable parameters into the Transformer, enhancing the expressive ability of positional encoding and thus improving detection accuracy. RT-DETR [16] designed an efficient hybrid backbone network, IoU-aware query selection, and multi-scale feature fusion strategies to achieve real-time and high-precision detection performance. Based on RT-DETR, RT-DETRv2 [19] sets different numbers of sampling points for features of different scales, enabling selective extraction of multi-scale features, and designs a dynamic data augmentation strategy to further improve object detection performance. Although the above methods have made progress based on the Transformer, they mainly focus on the enhancement and fusion of spatial-domain features. In contrast, our work extracts features from a multi-domain perspective and combines the Mamba structure to improve the performance of small object detection.

## 3. Method

**Figure 1:**
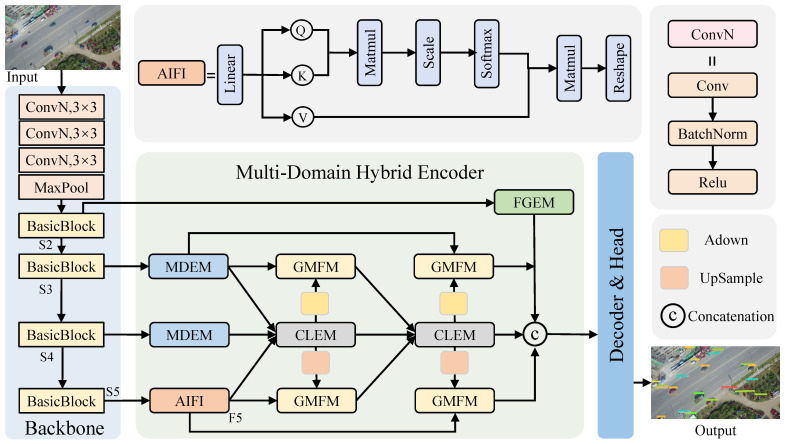
Overview of MDCL-DETR model. The model consists of four main parts: a backbone, multi-domain hybrid encoder, decoder and head. AIFI is a attention-based intra-scale feature interaction module.

The overall architecture of MDCL-DETR is illustrated in Figure 1, with RT-DETRv2 adopted as the baseline. MDCL-DETR is an end-to-end detector, consisting of a backbone network, a multi-domain hybrid encoder, a decoder, and a prediction head. The backbone network employs ResNet18, and the encoder comprises MDEM, CLEM, GMFM, and FGEM. Specifically, the input image is first fed into the backbone network for feature extraction. The extracted features are then transmitted to the multi-domain hybrid encoder, where multi-scale object features are extracted through multi-domain enhancement and cross-layer feature fusion. Subsequently, an IoU-aware query selection strategy is utilized to select features as the input to the decoder. Finally, the decoder performs iterative querying to generate accurate bounding boxes and corresponding confidence scores.

### 3.1. Multi-Domain Enhancement Module

**Figure 2:**
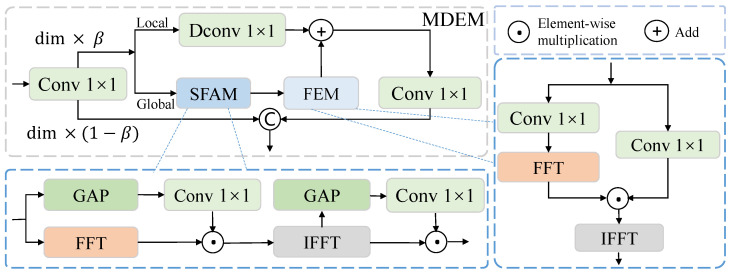
The structure of the MDEM. It uses a cross stage partial (CSP) structure to partition the frequency feature processing component, and the performs feature extraction via local and global branches.

Small objects in UAV imagery typically occupy only a small number of pixels, making their high-frequency details such as textures and edges highly susceptible to loss during downsampling. Meanwhile, complex backgrounds often exhibit strong textural interference, rendering it difficult to distinguish objects from clutter using spatial-domain features alone. The frequency domain, however, can explicitly separate high-frequency details from low-frequency background components, enabling both the preservation of the fine-grained information of small objects and the suppression of background noise. To address these issues, we propose MDEM, which achieves lightweight fusion of the spatial and frequency domains. This module enhances the high-frequency details of small objects while suppressing background clutter, thereby providing cleaner representations for subsequent feature extraction. The multi-domain enhancement module first utilizes the CSP (cross stage partial) structure to reduce the computational complexity of the model, and then combines the spatial and frequency domains to enhance detailed information and global structural features. The architecture of this module is illustrated in Figure 2, where dim denotes the dimension of the feature map. Specifically, for the input feature map $X\in R^{C\times H\times W}$, a convolutional layer (1 × 1) is used to adjust the number of channels, and then a division factor β is set to generate two parts:

(1) $$X_{\mathrm{MDEM}},X_{\mathrm{ident}}=\mathrm{Split}\left(X,\beta \right)$$

where $X_{\mathrm{MDEM}}\in R^{\beta \times C\times H\times W}$ denotes the feature component for subsequent multi-domain enhancement, and $X_{\mathrm{ident}}\in R^{(1-\beta )\times C\times H\times W}$ preserves the integrity of the original features. These two components are directly concatenated with the features enhanced by the MDEM to generate the following output features:

(2) $$X_{\mathrm{out}}=\mathrm{Concat}\left(\mathrm{Conv}\left(\mathrm{DConv}\left(X_{\mathrm{MDEM}}\right)+\mathrm{FEM}\left(\mathrm{SFAM}(X_{\mathrm{MDEM}})\right)\right)+X_{\mathrm{ident}}\right),$$

where Concat(·) denotes concatenation.

$X_{\mathrm{MDEM}}$ enhances the feature representation capability by fusing the spatial and frequency domains, and a local branch and a global branch are designed to strengthen local detailed features and global features, respectively. The local branch adopts depth-wise convolution to focus on extracting local detailed information in the spatial domain. The global branch consists of a spatial frequency attention module (SFAM) as well as a feature enhancement module (FEM), which fuses frequency-domain and spatial-domain features to enhance the network’s representation capability for small objects. Specifically, the feature $X_{\mathrm{MDEM}}$ is first fed into the global average, and then pooling is used to extract features and calculate the output frequency-domain attention weight. The frequency attention weight is then multiplied element-wise with the frequency-domain feature map obtained by the Fast Fourier Transform (FFT). Subsequently, the result is mapped back to the spatial domain via the Inverse Fast Fourier Transform (IFFT). The corresponding formula is as follows:

(3) $$X_{\mathrm{fd}}=IFFT\left(FFT\left(X_{\mathrm{MDEM}}\right)⊙Conv\left(\mathrm{GAP}\left(X_{\mathrm{MDEM}}\right)\right)\right)$$

where FFT(·) and IFFT(·) denote the Fast Fourier Transform and Inverse Fast Fourier Transform functions, respectively. Then, $X_{\mathrm{fd}}$ further optimizes the spatial-domain features and enhances the boundary and detailed information of the objects. The corresponding formula is as follows:

(4) $$X_{\mathrm{SFAM}}=X_{\mathrm{fd}}⊙Conv\left(\mathrm{GAP}(X_{\mathrm{fd}})\right)$$

Finally, $X_{\mathrm{SFAM}}$ is fed into the FEM. The FEM is divided into a frequency-domain branch and a spatial-domain branch, adopting a parallel structure to further enhance the object representation capability. In the frequency-domain branch, the input features are first processed by a 1 × 1 convolution layer, then transformed into frequency-domain features via Fourier Transform. In the spatial-domain branch, the input features are processed by a 1 × 1 convolution layer to generate spatial-domain features. Subsequently, the features generated by the frequency-domain branch and the spatial-domain branch are multiplied element-wise to obtain the multi-domain fused feature. Finally, they are mapped back to the spatial domain using the Inverse Fast Fourier Transform (IFFT). The corresponding formula is as follows:

(5) $$X_{\mathrm{FEM}}=\mathrm{IFFT}\left(\mathrm{Conv}\left(X_{\mathrm{SFAM}}\right)⊙\left(\mathrm{FFT}(\mathrm{Conv}\left(X_{\mathrm{SFAM}}\right))\right)\right)$$

### 3.2. Cross-Layer Feature Extraction Module

**Figure 3:**
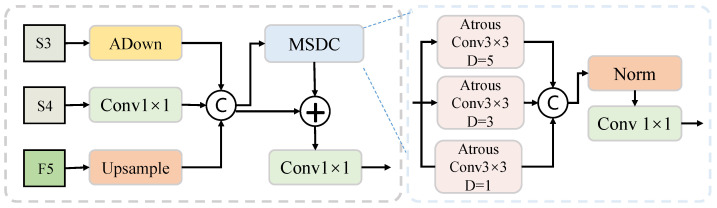
The structure of CLEM.

Shallow features (e.g., S3 layer) contain abundant detailed information, while high-level features (e.g., F5 layer) include high-level semantic information. The AIFI module performs intra-scale interaction on the S5 layer, which can capture the relationships between objects and facilitate object classification and localization. We design CLEM to complement detailed cues and semantic information through multi-scale feature fusion. It preserves the spatial location information of small objects and introduces global context, thereby improving detection stability under occlusion and dense scene conditions. The architecture of CLEM is illustrated in Figure 3. Specifically, S3 and F5 align the feature map scales with S4 through adaptive downsampling and upsampling, respectively. Then, the feature maps of the three scales (S3, S4, and F5) are concatenated and subsequently fed into a multi-scale dilated convolution (MSDC) block. This block captures multi-scale contextual information via multiple convolution layers with different dilation rates. Finally, the extracted features are transmitted to a 1 × 1 convolution layer to obtain the enhanced multi-scale features $X_{\mathrm{CLEM}}$.

(6) $$\begin{array}{cc} X_{\mathrm{CLEM}} & =\mathrm{Conv}\left(\mathrm{Concat}(\mathrm{AD}(S3),\mathrm{Conv}(S4),\mathrm{Up}(F5))\right)\\ \; & \;⊕\mathrm{MSDC}\left(\mathrm{Concat}(\mathrm{AD}(S3),\mathrm{Conv}(S4),\mathrm{Up}(F5)))\right) \end{array}$$

(7) $$\mathrm{MSDC}\left(F_{d=1},F_{d=2},F_{d=3}\right)=\mathrm{Conv}\left(\mathrm{Concat}\left(F_{d=1},F_{d=2},F_{d=3}\right)\right)$$

where AD(·) represents the adaptive downsampling operation, Up stands for the upsampling operation, and $F_{d=k}$ is the feature map extracted by atrous convolution with dilation rate *k*.

(8) $$\mathrm{VSSBlock}\left(F_{in}\right)=\mathrm{Linear}\left(\mathrm{SS}2D\left(\mathrm{SiLu}\left(\mathrm{DW}\;\mathrm{Conv}\left(\mathrm{Linear}\left(F_{in}\right)\right)\right)\right)\cdot \mathrm{Linear}\left(\mathrm{SiLu}\left(F_{in}\right)\right)\right)$$

### 3.3. Gated Mamba Feature Fusion Module

**Figure 4:**
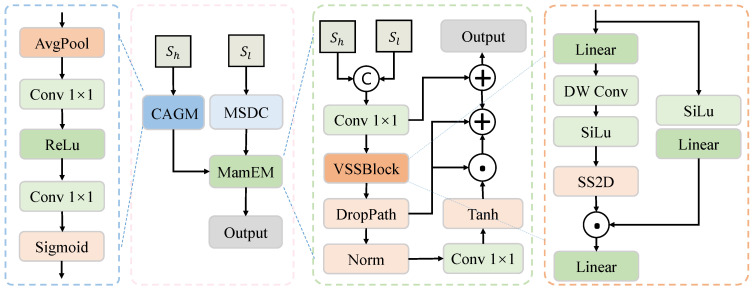
The structure of GMFM.

Multi-scale feature fusion inevitably suffers from feature redundancy and noise amplification. The gating mechanism can dynamically suppress irrelevant noise and enhance object-related features. Meanwhile, Mamba enables efficient long-range modeling and captures global dependency relationships among objects. Accordingly, we propose the GMFM to realize the dynamically weighted fusion of local details and global context, which further improves the feature discriminability of small objects. The architecture of GMFM is illustrated in Figure 4. This module integrates a channel attention gated module (CAGM), MSDC block, and Mamba feature enhancement module (MamEM), which adaptively correlates the local texture information of small objects with global scene information to improve the robustness of detection.

Specifically, the CAGM refines high-level semantic information, and the MSDC block also adopts convolutions with different dilation rates to capture rich features. The MamEM achieves efficient fusion of deep and shallow features by using the core structure of Visual Mamba, namely VSSBlock. Through the recursive update of state space, VSSBlock can accurately locate and enhance the local details of small objects under the guidance of global context while suppressing background interference and improving feature discriminability. The gated Mamba fusion feature $X_{\mathrm{GMFM}}$ can be obtained using the following calculation:

(9) $$X_{\mathrm{GMFM}}=\mathrm{MamEM}\left(\mathrm{CAGM}\left(S_{h}\right),\mathrm{MSDC}\left(S_{l}\right)\right)$$

(10) $$\mathrm{CAGM}\left(S_{h}\right)=\mathrm{sig}\left(\mathrm{Conv}\left(\mathrm{ReLu}\left(\mathrm{Conv}\left(\mathrm{AvgPool}(S_{h})\right)\right)\right)\right)$$

where $S_{h}$ denotes deep features and $S_{l}$ denotes shallow features, and sig(·) is the Sigmoid activation function. This operation adaptively weights feature channels to enhance object-related semantics and reduce background interference. In this case, DW Conv(·) is depth-wise convolution, SiLu(·) is the SiLu activation function, and SS2D(·) is the core component of Vision Mamba for long-range dependency modeling.

### 3.4. Fine-Grained Enhancement Module

**Figure 5:**
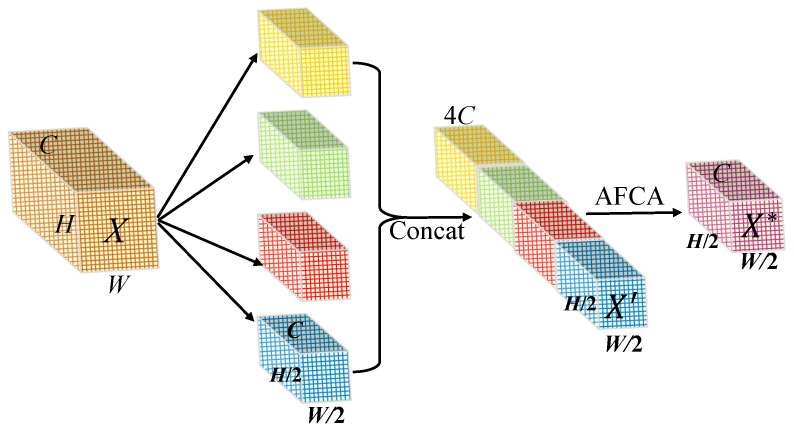
The structure of FGEM.

Small objects suffer from scarce pixels and blurred contours, and their fine-grained features are prone to attenuation during network propagation. Shallow features contain the most abundant edge and texture information. To address this issue, we propose a fine-grained enhancement module (FGEM). Through spatial feature reorganization and adaptive attention, the proposed module strengthens and compensates for shallow detailed cues, recovers the edge and texture information of small objects, and ultimately improves their identifiability. The architecture of FGEM is illustrated in Figure 5.

The P2 layer contains rich detailed information of small objects, and FGEM takes the P2 feature map as input, $X\in R^{C\times H\times W}$, where *C*, *H*, and *W* denote the number of channels, height, and width of the feature map, respectively. To preserve more fine-grained information of small objects, FGEM first processes the input feature map through spatial feature reorganization. Specifically, the input feature map is divided into four sub-feature maps, and features are extracted according to the combination of odd/even rows and columns using the following formula:

(11) $$X_{\mathrm{even},\mathrm{even}}=X\left[:,0:H:2,0:W:2\right],X_{\mathrm{even},\mathrm{odd}}=X\left[:,0:H:2,1:W:2\right]$$

(12) $$X_{\mathrm{odd},\mathrm{even}}=X\left[:,1:H:2,0:W:2\right],X_{\mathrm{odd},\mathrm{odd}}=X\left[:,1:H:2,1:W:2\right]$$

where $X_{\mathrm{even},\mathrm{even}}$, $X_{\mathrm{even},\mathrm{odd}}$, $X_{\mathrm{odd},\mathrm{even}}$, and $X_{\mathrm{odd},\mathrm{odd}}$ denote the sub-feature maps obtained from the input feature map under different sampling modes, respectively. Subsequently, these sub-feature maps are concatenated along the channel dimension to generate a new feature map, as follows:

(13) $$X^{\prime }=\mathrm{Concat}\left(X_{\mathrm{even},\mathrm{even}},X_{\mathrm{even},\mathrm{odd}},X_{\mathrm{odd},\mathrm{even}},X_{\mathrm{odd},\mathrm{odd}}\right)$$

where $X^{\prime }\in R^{4C\times \frac{H}{2}\times \frac{W}{2}}$ denotes the concatenated feature map. The number of channels in the feature map is expanded to four times the original, while the spatial size is reduced to one-fourth of the original, effectively preserving more fine-grained information. Subsequently, the Attention Fusion and Channel Attention (AFCA) mechanism is adopted for further enhancement. Through global average pooling and convolution, global and detailed information is obtained. Learnable parameters are introduced to dynamically adjust the importance of global and local information, suppress useless features, and enhance the fine-grained features of small objects.

(14) $$\mathrm{AFCA}\left(X^{\prime }\right)=X^{\prime }\cdot \mathrm{sig}\left(\mathrm{Conv}1d\left(\gamma \cdot A_{1}+\left(1-\gamma \right)\cdot A_{2},W_{1}\right)\right)$$

(15) $$A_{1}=\mathrm{sig}\left(\mathrm{sum}\left(\mathrm{Conv}1d\left(\mathrm{AvgPool}(X^{\prime })\right)\right)\cdot \mathrm{Conv}2d\left(\mathrm{AvgPool}(X^{\prime })\right)\right)$$

(16) $$A_{2}=\mathrm{sig}\left(\mathrm{sum}\left(\mathrm{Conv}2d\left(\mathrm{AvgPool}(X^{\prime })\right)\right)\cdot \mathrm{Conv}1d\left(\mathrm{AvgPool}(X^{\prime })\right)\right)$$

where γ is a learnable factor, and sig(·) denotes sigmoid activation operation.

### 3.5. Loss Function

In this paper, with reference to the RT-DETRv2 loss, Focal Loss is adopted as the classification loss, and GIoU loss is selected as the regression loss. The total loss is the weighted sum of the classification loss and the regression loss, and its specific expression is as follows:

(17) $$L_{\mathrm{total}}=\sum_{i=1}^{N}{⊮}_{\left\{c_{\sigma (i)}\neq \emptyset \right\}}\left[L_{\mathrm{Focal}}\left({\hat{p}}_{i},c_{\sigma (i)}\right)+\lambda \cdot L_{\mathrm{GIoU}}\left({\hat{b}}_{i},b_{\sigma (i)}\right)\right]$$

In this formula, ${⊮}_{\left\{\cdot \right\}}$ is an indicator function, which takes a value of 1 when $c_{\sigma (i)}\neq \emptyset$ (i.e., the query is matched to a ground-truth object) and 0 otherwise, so that the loss is only calculated for positive samples; $L_{\mathrm{Focal}}$ is the Focal Loss for classification tasks, whose expression is as follows:

(18) $$L_{\mathrm{Focal}}\left({\hat{p}}_{i},c_{\sigma (i)}\right)=-\alpha {\left(1-{\hat{p}}_{i}\left(c_{\sigma (i)}\right)\right)}^{\mu }\log{\hat{p}}_{i}\left(c_{\sigma (i)}\right)$$

where α is the class balance coefficient, μ is the focusing parameter, and ${\hat{p}}_{i}\left(c_{\sigma (i)}\right)$ is the probability that the *i*-th query is predicted to be the matched class $c_{\sigma (i)}$; $L_{\mathrm{GIoU}}$ is the GIoU loss for bounding box regression, which measures the spatial overlap and position deviation between the predicted box ${\hat{b}}_{i}$ and the ground-truth box $b_{\sigma (i)}$; and λ is the regression loss weight, which is used to balance the contributions of the classification loss and the regression loss.

## 4. Results

### 4.1. Implementation Details

All experiments were conducted on a Ubuntu 18.04 system, an NVIDIA Tesla V100 GPU (Nvidia Corporation, SantaClara, CA, USA), and an Intel(R) Xeon(R) CPU E5-2650 processor (Intel Corporation, SantaClara, CA, USA). The software environment includes Python 3.8, Pytorch 2.0, and CUDA 12.1. Our experiments adopt RT-DETRv2-S (Baidu Inc., Beijing, China) and the pre-trained ResNet-18 as the baseline model. The AdamW optimizer [35] is used, with the learning rate and weight decay coefficient both set to 0.0001, the batch size set to 4, and the total number of training epochs set to 400. The division factor β is set to 0.25.

### 4.2. Dataset and Evaluation Metrics

To verify the effectiveness of our proposed method, extensive experiments are conducted on two benchmark datasets for aerial object detection. Our experiments are mainly based on the VisDrone2019 dataset [36], which is a drone-view object detection dataset covering various environments such as complex urban scenes, dense crowds, and transportation hubs. It consists of 6471 training images, 548 validation images, and 3190 test images, including 10 categories such as pedestrian, person, bicycle, car, van, truck, tricycle, awning-tricycle, bus, and motor. To evaluate the generalization ability and robustness of our method, we perform an extended evaluation on the challenging AI-TOD dataset [37]. AI-TOD is a large-scale and highly challenging benchmark dataset specifically designed for tiny object detection in aerial imagery, focusing on pixel-level extremely small objects (with an average size of only 12.8 pixels). It contains 11,214 training images, 2804 validation images, and 14,018 test images, covering eight categories, including airplane, bridge, storage tank, ship, swimming pool, vehicle, person, and wind mill. To evaluate the performance of our method, we adopt the MS COCO standard for evaluation, including mAP_50–95_, mAP_50_, parameters (Params), and giga floating-point operations (GFLOPs). AP (average precision) is the evaluation metric for a single category, calculated by computing precision (P) and recall (R) at different confidence thresholds; a higher AP value indicates better detection performance of the model for that category. The corresponding formula is as follows:

(19) $$Precision=\frac{TP}{TP+FP}$$

(20) $$Recall=\frac{TP}{TP+FN}$$

(21) $$AP=\int_{0}^{1}P\left(r\right)dr$$

where TP, FP, and FN represent true positives, false positives, and false negatives, respectively. P(r) denotes the precision at a recall threshold of *r*. The mAP refers to the mean average precision across all categories, which is used to measure the overall detection performance of the model. Specifically, mAP_50_ is the mAP at an IoU threshold of 0.5, while mAP_50–95_ is the average mAP across IoU thresholds ranging from 0.5 to 0.95 with an interval of 0.05. Params indicates model size, and GFLOPs measures computational complexity. To further demonstrate the performance in terms of object size, we also use ${\mathrm{AP}}_{S}$, ${\mathrm{AP}}_{M}$, and ${\mathrm{AP}}_{L}$ on COCO. ${\mathrm{AP}}_{S}$, ${\mathrm{AP}}_{M}$, and ${\mathrm{AP}}_{L}$ are the AP values for small, medium, and large objects of different scales, respectively. The MS COCO dataset defines object scales based on bounding box area: objects with an area smaller than 32×32 pixels are classified as small, those between 32×32 and 96×96 pixels as medium, and those larger than 96×96 pixels as large. For the AI-TOD dataset, considering the tiny size of aerial objects, Wang et al. [37] further categorize object scales into four levels: very tiny, tiny, small, and medium. In accordance with the official definition of AI-TOD, objects covering 2–8 pixels are defined as very tiny objects, those within 8–16 pixels as tiny objects, 16–32 pixels as small instances, and 32–64 pixels as medium instances. Notably, this dataset contains no large-sized objects. Consequently, we adopt ${\mathrm{AP}}_{vt}$, ${\mathrm{AP}}_{t}$, ${\mathrm{AP}}_{s}$, and ${\mathrm{AP}}_{m}$ to quantitatively evaluate and present the detection performance.

### 4.3. Comparative Experiments

To demonstrate our excellent detection performance, we conducted extensive comparative experiments on the VisDrone2019 dataset between our algorithm and various state-of-the-art object detection algorithms, including CNN-based object detection algorithms (e.g., Faster R-CNN, RetinaNet, YOLOv10, YOLO11, etc.) and Transformer-based object detection algorithms (e.g., Deformable DETR, DINO, RT-DETR, etc.). The corresponding experimental results are presented in Table 1. (Note: “-” indicates that the performance of the method was not verified on the corresponding metric).

As can be observed from the experimental results in Table 1, the YOLO-series algorithms achieve a high inference speed but are slightly insufficient in detection accuracy. The Transformer-based DETR-series methods can focus on the key regions of objects, leading to improved accuracy, yet require a larger number of parameters. The proposed MDCL-DETR algorithm adopts multi-feature fusion to enhance feature representation. Specifically, compared with the large-scale YOLO11 model, MDCL-DETR improves mAP_50_ by 11.2% with similar parameters. Compared with the baseline RT-DETRv2-S, our MDCL-DETR-S improves mAP_50_ by 4.8% and ${\mathrm{AP}}_{S}$ by 4.2%, while introducing 5.6M additional parameters. Meanwhile, compared with Van-DETR and UAV-DETR, MDCL-DETR achieves 6.5% and 5.5% improvements in mAP_50_, respectively. These experimental results demonstrate that the proposed MDCL-DETR outperforms the comparison methods on multiple evaluation metrics. The complementarity of multi-domain features and cross-layer fusion can effectively improve the detection performance of small objects and occluded objects.

To further verify the generalization ability of the MDCL-DETR model, we evaluate our method on the AI-TOD dataset, with the experimental results presented in Table 2. The experimental results demonstrate that the proposed MDCL-DETR outperforms the baseline RT-DETRv2 across multiple evaluation metrics, achieving consistent performance gains on both tiny and small objects. In particular, MDCL-DETR obtains performance improvements of 3.1% and 2.0% for tiny and small object detection, respectively. This confirms that our method can effectively enhance the detailed feature representation of objects via multi-scale feature fusion and further boost overall detection performance.

### 4.4. Ablation Study

To verify the effectiveness of the key modules in the proposed method, ablation experiments are designed in this section for its key components, including the multi-domain enhancement module, cross-layer feature extraction module, gated Mamba feature fusion module, and fine-grained enhancement module. All experiments are conducted on the public VisDrone2019 dataset for performance evaluation, enabling systematic analysis of the contribution of each component to object detection performance.

#### 4.4.1. Ablation Analysis of Different Modules

To analyze the effects of the multi-domain enhancement module, cross-layer feature extraction module, gated Mamba feature fusion module, and fine-grained enhancement module on detection performance, ablation experiments are conducted on the VisDrone2019 dataset, with the results shown in Table 3. Compared with the baseline model, introducing the multi-domain enhancement module (MDEM) improves the model’s mAP_50_ from 49.3% to 52.4% and ${\mathrm{AP}}_{S}$ from 21.1% to 23.4%, indicating that incorporating frequency-domain features can effectively strengthen the model’s ability to capture high-frequency detailed information. When only the cross-layer feature extraction module (CLEM) is used, the model achieves a mAP_50_ of 50.1% and ${\mathrm{AP}}_{S}$ of 21.4%, demonstrating that fusing features from different layers enhances the object representation capability. When MDEM is combined with CLEM, mAP_50_ increases to 53.5% and ${\mathrm{AP}}_{S}$ rises to 24.3%. This shows that the spatial frequency domain enhancement achieved by MDEM provides a higher-quality feature foundation for subsequent cross-layer feature fusion in CLEM. When using only the gated Mamba feature fusion module (GMFM) and the fine-grained enhancement module (FGEM), mAP_50_ improves by 1.2% and 2.2%, respectively, indicating that effectively capturing object dependencies and compensating with shallow features is beneficial for improving small object detection performance. Finally, when all modules are integrated, compared with the baseline method, the model’s mAP_50_ increases from 49.3% to 54.1% and ${\mathrm{AP}}_{S}$ rises from 21.1% to 24.6%. The experimental results show that different layers of the network contain differentiated feature information, and effectively enhancing and fusing multi-layer features can significantly strengthen the object representation capability. (Note: “√” indicates that the corresponding column module is adopted by the model in the experiment.).

#### 4.4.2. Ablation Analysis of the MDEM

To analyze the effect of the multi-domain enhancement module on object detection performance, ablation experiments are conducted by disassembling its global branch and local branch, with the experimental results shown in Table 4. These data indicate that when adding the local branch, mAP_50_ and ${\mathrm{AP}}_{S}$ are improved to 49.7% and 21.5%, respectively, verifying that the local branch can enhance the ability to extract local detailed information. With the addition of the SFAM, mAP_50_ and ${\mathrm{AP}}_{S}$ increase to 50.5% and 22.0%, respectively, which demonstrates that the SFAM can effectively enhance the representation capability of small objects by guiding frequency-domain feature weighting through spatial localization. With the introduction of the multi-domain enhancement module, mAP_50_ and ${\mathrm{AP}}_{S}$ are finally increased to 52.4% and 23.4%, illustrating that the MDEM strengthens the object representation capability by fusing frequency-domain and spatial-domain features.

#### 4.4.3. Ablation Analysis of the GMFM

To analyze the effect of the gated Mamba feature fusion module (GMFM) on detection performance, ablation experiments are conducted on the number of GMFMs used, with the experimental results shown in Table 5. UL, UR, LL, and LR represent the positions of the GMFM in the model, where UL corresponds to the upper left position, UR denotes the upper right position, LL indicates the lower left position, and LR stands for the lower right position, as illustrated in the Figure 1. These experimental results show that after adding the UL module, mAP_50_ and ${\mathrm{AP}}_{S}$ are improved to 49.4% and 21.1%, respectively, indicating that the multi-scale features generated by the UL module preserve more detailed object information. After adding the UR module, mAP_50_ and ${\mathrm{AP}}_{S}$ increase to 50.4% and 21.8%, respectively, demonstrating that the UR module can effectively capture global semantic information by fusing features from different dimensions. When both the LL and LR modules are integrated, mAP_50_ and ${\mathrm{AP}}_{S}$ are further improved to 51.1% and 22.4%, respectively, which illustrates that feature fusion across different layers with Mamba enhances the ability to extract both global and local information, thus effectively boosting the detection performance of the model.

### 4.5. Visualization

**Figure 6:**
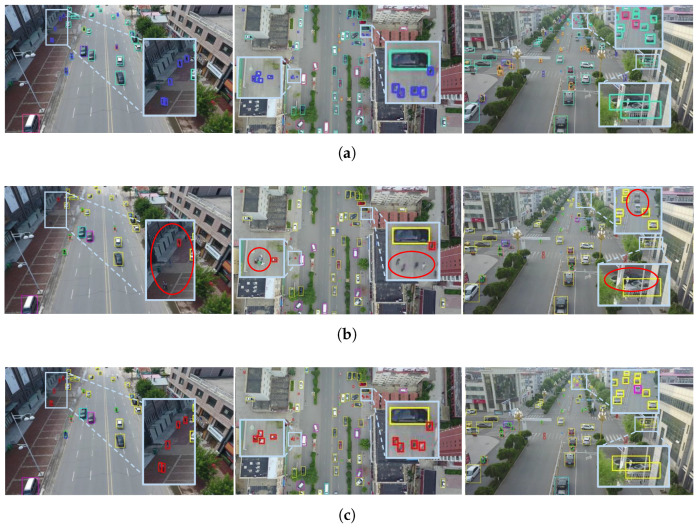
Visualization of detection results on the Visdrone dataset. Each column displays (**a**) the ground truth, (**b**) the baseline’s (RT-DETRv2-S) result, and (**c**) our model’s (MDCL-DETR) result. The red circles in (**b**) denote the missed-detection regions, which are capable of detecting objects successfully by our model in (**c**).

**Figure 7:**
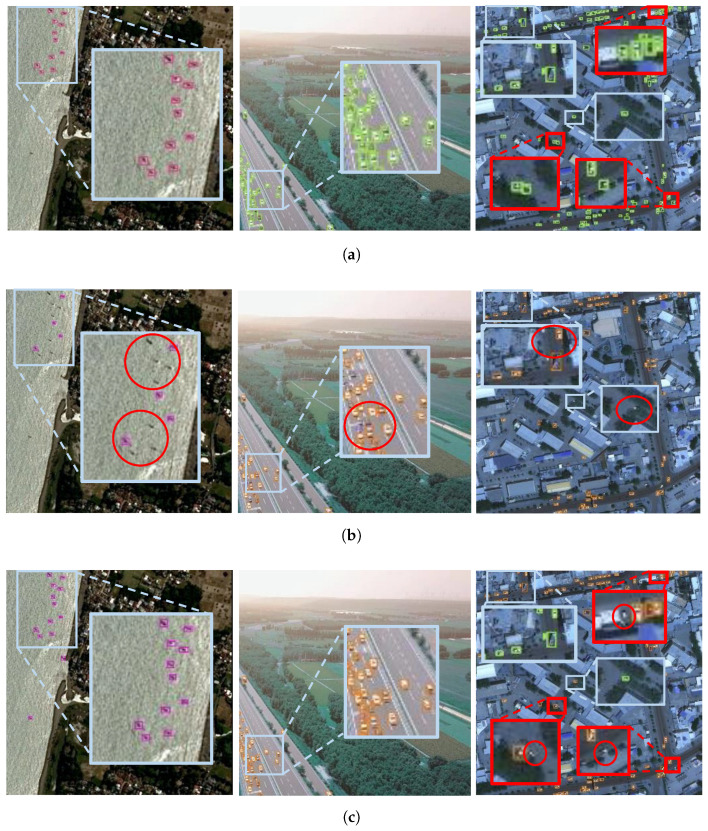
Visualization of detection results on the AI-TOD dataset. (**a**) Ground truth, (**b**) the baseline’s (RT-DETRv2-S) result, and (**c**) our model’s (MDCL-DETR) result. The visualization results demonstrate that compared with the baseline model, our method can effectively detect small objects and reduce missed detections.

**Figure 8:**
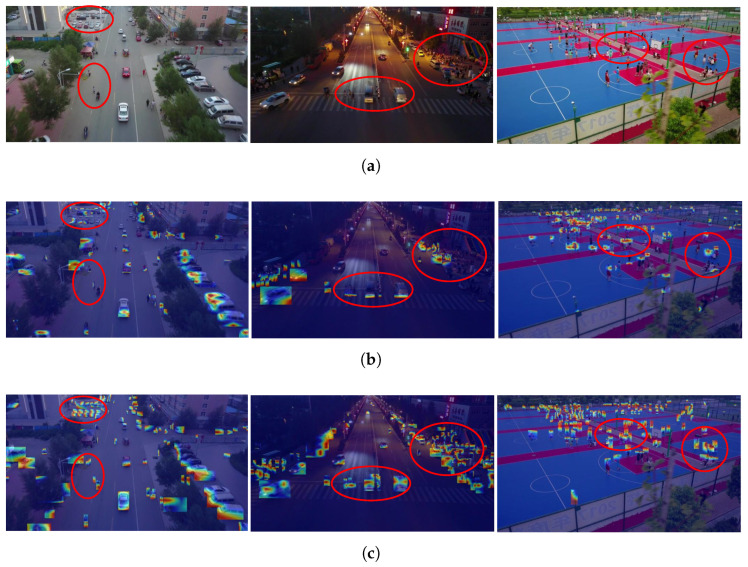
Visualization of attention heatmaps on the VisDrone2019 dataset. (**a**) Original image, (**b**) attention heatmap of the baseline model RT-DETRv2-S, and (**c**) attention heatmap of the proposed MDCL-DETR model. The results demonstrate that our method can concentrate highlights on the object regions.

To qualitatively evaluate the detection performance, we visualize the detection results and heatmaps of the baseline RT-DETRv2-S and the proposed MDCL-DETR on the VisDrone and AI-TOD datasets, as illustrated in Figure 6, Figure 7, and Figure 8, respectively. Compared with RT-DETRv2-S, MDCL-DETR can accurately detect small objects and occluded objects, and exhibits more concentrated and precise attention on small objects.

Figure 6 presents a comparison of partial visualized detection results between the proposed method (MDCL-DETR) and the baseline model RT-DETRv2-S on the VisDrone2019 dataset. Specifically, Figure 6a shows the ground-truth results, while Figure 6b and Figure 6c display the detection results of RT-DETRv2-S and MDCL-DETR, respectively. It can be observed from the results that MDCL-DETR exhibits superior detection capability in complex scenarios. As highlighted by the red circles in Figure 6b, RT-DETRv2-S suffers from missing detections, whereas MDCL-DETR can not only accurately detect small-scale pedestrian objects but also effectively identify occluded pedestrians and vehicles. These results fully demonstrate that MDCL-DETR has significant advantages in small object detection and occluded object handling.

Figure 7 displays the model output results of different scenarios selected from the AI-TOD dataset. Figure 7a shows the ground-truth results, while Figure 7b and Figure 7c display the detection results of RT-DETRv2-S and MDCL-DETR, respectively. Our method can not only identify dense small objects but also detect objects that are not present in the ground-truth annotations, demonstrating the effectiveness and generalization of our algorithm in small object detection. Furthermore, the red boxed region in the third column of Figure 7c shows a missed-detection case. As can be observed from the zoomed-in details, the missed target is an object with extremely few pixels and weak features, which is easily confused with complex backgrounds such as building shadows and vegetation textures.

To further analyze the contribution of the proposed improved structure to the performance improvement in the model, we select images from different scenarios in the VisDrone2019 dataset and use Grad-CAM to visualize the heatmaps of intermediate features for different models. Figure 8a shows the images in different scenarios during daytime and nighttime. Figure 8b and Figure 8c present the heatmap visualization results of the baseline model RT-DETRv2-S and our MDCL-DETR model, respectively, where the red regions indicate the significant attention areas of the model.

As shown in Figure 8b, for small objects in the distance at the edge of the court, the baseline model exhibits vague attention regions, making it difficult to accurately locate the contours of small objects. For occluded objects in crowds, the response intensity is insufficient, and it is challenging to distinguish objects from the background, showing a sparse and localized activation pattern. This indicates that the baseline model pays insufficient attention to the local details of small objects and lacks contextual modeling of spatial and occlusion relationships between objects. As shown in Figure 8c, MDCL-DETR can accurately activate small human objects with higher activation intensity and clear boundaries. In scenes with mutual occlusion between humans, it can still clearly activate the key regions of each occluded object, focusing on the object body rather than the background. This demonstrates that our method can effectively capture the key features and semantic information of objects.

### 4.6. Inference Speed and Deployment Evaluation

We evaluate the inference speed of MDCL-DETR-S and MDCL-DETR-M on three typical hardware platforms, as shown in Table 6. On the Jetson Xavier NX (NVIDIA Corporation, SantaClara, CA, USA), MDCL-DETR-S achieves 20 FPS, exceeding the 10 FPS real-time requirement for UAV applications. MDCL-DETR-M reaches 13 FPS on the same platform, offering a good balance between accuracy and speed. These results confirm that the proposed method meets the real-time processing constraints of embedded edge devices, demonstrating its practical value for UAV-borne visual sensing systems.

## 5. Conclusions

In this paper, we propose MDCL-DETR, an innovative DETR-based method for small object detection in aerial images, aiming to address the difficulty of extracting discriminative representations for small objects. We design four specialized modules (MDEM, CLEM, GMFM, and FGEM) to progressively extract and refine multi-scale features, thereby significantly enhancing detection performance. The MDEM enhances object details and global structures, distinguishes object features from background interference, and lays a high-quality feature foundation for subsequent processing. The CLEM aggregates cross-layer multi-scale features, preserves the spatial details of small objects while integrating high-level contextual semantics, and alleviates information loss caused by downsampling. The GMFM models long-range dependencies of multi-scale features, realizes dynamic fusion of local details and global context, and further improves feature discriminability and global modeling capability. The FGEM reinforces and compensates fine-grained features, ensuring that critical small-object details are retained in the final feature representation. Extensive experimental results demonstrate that MDCL-DETR achieves excellent performance in small aerial object detection tasks. On the VisDrone2019 dataset, compared with the baseline method, MDCL-DETR improves the mAP_50_ by 4.8% and the ${\mathrm{AP}}_{S}$ by 3.9%. Although significant progress has been made in this work for small object detection, the proposed method still suffers from missed detections on extremely tiny objects, and its computational efficiency remains inferior to that of single-stage detectors. In future research, we will further explore lightweight network designs and dynamic inference strategies to improve both the detection accuracy of extremely tiny objects and the model’s inference speed, thereby making the model better suited for real-time applications such as UAV aerial imagery and remote sensing object detection.

## Figures and Tables

**Table 1:** Comparison of different object detection methods on the VisDrone2019 dataset.

Method	mAP_50–95_	mAP_50_	${\mathrm{AP}}_{S}$	${\mathrm{AP}}_{M}$	${\mathrm{AP}}_{L}$	Params (M)	GFLOPs	FPS
Faster R-CNN [38] (2015)	23.6%	39.1%	14.8%	34.8%	40.8%	41.4	208	55
Cascade R-CNN [39] (2018)	23.6%	39.1%	14.8%	35.3%	41.8%	69.3	236	45
RetinaNet [40] (2017)	18.0%	38.2%	8.2%	30.4%	37.2%	36.5	210	63
TOOD [41] (2021)	23.7%	39.2%	14.7%	35.0%	42.6%	32.0	199	44
YOLOv10-l [7] (2024)	26.5%	43.5%	16.6%	39.3%	47.4%	25.7	126	150
YOLOv11-l [8] (2024)	26.2%	42.9%	16.5%	38.8%	48.5%	25.3	87	45
YOLOv12-l [9] (2024)	26.4%	43.3%	16.4%	39.4%	47.3%	26.3	89	42
PPM-YOLOv11 [5] (2026)	24.6%	40.9%	-	-	-	19.6	29	-
Deformable DETR [14] (2020)	27.1%	42.2%	19.1%	34.4%	40.2%	40.0	173	29
Sparse DETR [42] (2021)	27.3%	42.5%	-	35.0%	40.2%	41.0	121	35
DINO [43] (2022)	29.4%	46.2%	22.9%	38.4%	44.5%	47.6	279	21
RT-DETR [16] (2024)	27.1%	45.0%	19.1%	37.1%	42.5%	19.9	57	90
Van-DETR [44] (2025)	28.1%	47.6%	-	-	-	38.0	127	98
UAV-DETR [45] (2025)	29.8%	48.6%	-	-	-	20.0	77	54
HF-DETR [46] (2026)	29.0%	47.4%	-	-	-	8.6	37	121
RT-DETRv2-S [19] (2024)	29.4%	49.3%	20.4%	40.5%	51.5%	20.0	60	198
RT-DETRv2-M [19] (2024)	30.3%	51.1%	20.4%	42.8%	59.9%	34.0	136	109
MDCL-DETR-S (ours)	33.1%	54.1%	24.6%	43.9%	53.9%	25.6	147	83
MDCL-DETR-M (ours)	33.2%	54.1%	24.3%	44.7%	55.7%	38.0	186	60

**Table 2:** Comparison of different object detection methods on the AI-TOD dataset.

Method	mAP_50–95_	mAP_50_	${\mathrm{AP}}_{vt}$	${\mathrm{AP}}_{t}$	${\mathrm{AP}}_{s}$	${\mathrm{AP}}_{m}$
Faster R-CNN [38] (2015)	11.3%	25.4%	0.0%	7.2%	23.3%	33.6%
Cascade R-CNN [39] (2018)	13.5%	29.1%	0.0%	10.5%	25.5%	36.6%
NWD-RKA [47] (2022)	23.4%	53.5%	8.7%	23.8%	28.5%	36.0%
HANet [48] (2023)	22.1%	53.7%	10.9%	22.2%	27.3%	36.8%
MENet [49] (2024)	23.2%	56.2%	9.7%	23.9%	25.3%	34.4%
LTDNet [30] (2025)	23.0%	54.6%	8.9%	23.6%	27.2%	33.1%
RT-DETRv2-S [19] (2024)	23.8%	51.3%	10.1%	24.2%	29.5%	37.7%
MDCL-DETR-S (Ours)	26.8%	56.2%	12.2%	27.1%	31.5%	40.4%

**Table 3:** Ablation study of key modules.

MDEM	CLEM	GMFM	FGEM	mAP_50–95_	mAP_50_	${\mathrm{AP}}_{S}$	${\mathrm{AP}}_{M}$	${\mathrm{AP}}_{L}$	Recall	Params/M
				29.4%	49.3%	21.1%	39.8%	51.0%	45.6%	20.0
√				31.8%	52.4%	23.4%	42.5%	53.5%	47.4%	20.7
	√			29.7%	50.1%	21.4%	40.1%	48.9%	46.1%	19.8
		√		30.8%	51.1%	22.3%	41.3%	52.1%	46.8%	24.2
			√	31.7%	52.0%	23.3%	42.2%	54.0%	47.4%	21.0
√	√			32.5%	53.5%	24.3%	43.1%	52.4%	48.1%	20.7
√	√	√		32.8%	53.7%	24.5%	43.4%	52.7%	48.3%	24.7
√	√	√	√	33.1%	54.1%	24.6%	43.9%	53.9%	48.3%	25.6

**Table 4:** Ablation study of MDEM.

Local	Global		${\mathrm{mAP}}_{50–95}$	${\mathrm{mAP}}_{50}$	${\mathrm{AP}}_{S}$	${\mathrm{AP}}_{M}$	${\mathrm{AP}}_{L}$	Recall	Params/M
	SFAM	FEM							
			29.4%	49.3%	21.1%	39.8%	51.0%	45.6%	20.0
√			30.2%	49.7%	21.5%	41.0%	50.0%	46.0%	20.4
	√		30.6%	50.5%	22.0%	41.3%	50.9%	46.5%	20.4
		√	30.4%	50.0%	21.9%	41.0%	51.7%	46.5%	20.4
√	√		31.3%	51.5%	22.6%	42.1%	52.1%	47.1%	20.5
√	√	√	31.8%	52.4%	23.4%	42.5%	53.7%	47.6%	20.7

**Table 5:** Ablation study of GMFM.

UL	UR	LL	LR	mAP_50–95_	mAP_50_	${\mathrm{AP}}_{S}$	${\mathrm{AP}}_{M}$	${\mathrm{AP}}_{L}$	Recall	Params/M
				29.4%	49.3%	21.1%	39.8%	51.0%	45.6%	20.0
√				29.9%	49.4%	21.1%	40.5%	52.4%	45.9%	21.0
	√			30.0%	49.5%	21.2%	40.7%	52.7%	45.8%	20.8
		√		30.1%	49.6%	21.3%	41.0%	51.8%	46.1%	21.0
			√	30.3%	49.8%	21.6%	41.0%	49.4%	46.1%	20.8
√	√			30.6%	50.4%	21.8%	41.5%	51.3%	46.5%	21.8
√	√	√		30.9%	50.9%	22.2%	41.5%	51.2%	46.7%	23.4
√	√	√	√	31.1%	51.1%	22.4%	41.9%	52.9%	46.9%	24.2

**Table 6:** Inference speed of MDCL-DETR on different deployment hardware.

Model	NVIDIA Tesla V100	Jetson Xavier NX	Intel(R) Xeon(R) E5-2650 CPU
MDCL-DETR-S	89 FPS	20 FPS	15 FPS
MDCL-DETR-M	65 FPS	13 FPS	9 FPS

## Data Availability

The VisDrone2019 dataset used in this study is publicly available at the following official repository: https://github.com/VisDrone/VisDrone-Dataset (accessed on 30 March 2026). The AI-TOD dataset used in this study is publicly available at the following official repository: https://github.com/jwwangchn/AI-TOD (accessed on 30 March 2026).
